# Estabilidad de la alanina aminotransferasa en suero. Efecto del modelo de estudio, la concentración catalítica inicial y la plataforma analítica

**DOI:** 10.1515/almed-2019-0029

**Published:** 2020-04-08

**Authors:** Josep Miquel Bauça, Andrea Caballero, Carolina Gómez, Débora Martínez-Espartosa, Isabel García del Pino, Juan José Puente, Maria Antonia Llopis, Itziar Marzana, Marta Segovia, Mercedes Ibarz, Montserrat Ventura, Paloma Salas, Rubén Gómez-Rioja

**Affiliations:** Servicio de Anàlisis Clínicos, Hospital Universitari Son Espases, Ctra. de Valldemossa, 79, module J+1, 07120, Palma, Illes Balears, Spain; Comisión de Calidad Extraanalítica, SEQC-ML, Viladecans, Spain; Servicio de Bioquímica Clínica, Laboratorios Clínicos, Hospital Universitario Vall d’Hebron, Barcelona, Spain; Servicio de Análisis y Bioquímica Clínica, Laboratori Clínic Metropolitana Nord, Hospital Universitario Germans Trias i Pujol, Badalona, Spain; Laboratorio de Área, Complexo Hospitalario Universitario, A Coruña, Spain; Unidad Extraanalítica, Laboratorios Hospital Universitario Cruces, Baracaldo (Vizcaya), Spain; Servei d’Anàlisis Clíniques, Hospital Universitari Arnau de Vilanova, IRBLleida, Lleida, Spain; Programas de Garantía Externa de la Calidad, Sociedad Española de Medicina de Laboratorio (SEQCML), Barcelona, Spain; Servicio de Análisis Clínicos, Hospital La Paz-Cantoblanco-Carlos III, Madrid, Spain

**Keywords:** alanina aminotransferasa (ALT), conservación, enzima, estabilidad, preanalítica

## Abstract

**Objetivos:**

Existen multitud de estudios de estabilidad de magnitudes de interés clínico, aunque con resultados muy diferentes o incluso contradictorios, como es el caso de la alanina aminotransferasa (ALT). El objetivo de este estudio fue evaluar la estabilidad de la ALT en suero incluyendo múltiples variables.

**Métodos:**

Se realizó un estudio multicéntrico en ocho laboratorios, con muestras de suero con concentración catalítica inicial conocida de ALT, en cuatro intervalos diferentes: <50 U/L (<0.83 μkat/L), 50–200 U/L (0.83–3.33 μkat/L), 200–400 U/L (3.33–6.67 μkat/L) y >400 U/L (>6.67 μkat/L). Se conservaron las muestras durante siete días a dos temperaturas diferentes, siguiendo cuatro modelos experimentales y utilizando cuatro plataformas analíticas diferentes. Se calcularon las respectivas ecuaciones de estabilidad mediante regresión lineal y se valoró la influencia de las diferentes variables en un modelo multivariante.

**Resultados:**

Se observó una disminución constante de la concentración catalítica de la ALT respecto al tiempo. Asimismo, se observó un marcado efecto de la temperatura y de la plataforma analítica utilizada (−4%/día a temperatura ambiente frente a −1%/día refrigerado), con una mayor inestabilidad en Architect (Abbott). Se detectó un ligero efecto de la concentración catalítica inicial de ALT, sin embargo, no se observó influencia del modelo experimental utilizado.

**Conclusiónes:**

La ALT en suero sufre una disminución constante en el tiempo que se mitiga con la refrigeración de la muestra. Se ha encontrado un efecto significativo de variables poco estudiadas que, unidas a una gran variabilidad interindividual, hacen necesarios estudios con un alto número de muestras para determinar las ecuaciones de estabilidad.

## Introducción

Para la correcta interpretación de los resultados de una prueba de laboratorio, la composición de las muestras analizadas *in vitro* debe reflejar el valor real *in vivo*, lo que supone que la concentración de cualquier componente no debería cambiar durante la fase preanalítica. La estabilidad de las magnitudes bioquímicas se define como la capacidad de una muestra de conservar el valor inicial de las propiedades dentro de unos límites establecidos durante un determinado periodo de tiempo cuando esta se mantiene en unas determinadas condiciones [1, 2]. Se han llevado a cabo multitud de estudios de estabilidad para los parámetros más comunes medidos en suero y plasma en los laboratorios clínicos en diversas condiciones, pero con resultados muy diferentes o incluso discrepantes. Esto es debido a la falta de diseños experimentales estandarizados, a la ausencia de guías o recomendaciones firmes sobre la forma en la que los estudios de estabilidad deberían llevarse a cabo y a la amplia variabilidad de especificaciones de inestabilidad máxima permisible [3–5]. En 2006 la Sociedad Española de Medicina de Laboratorio (SEQC^ML^) abordó este problema y desarrolló un protocolo de evaluación de la estabilidad. En 2019 se ha publicado la segunda versión, que incluye un estudio preliminar y un estudio de estabilidad completo que permite definir la ecuación de estabilidad [1].

La alanina aminotransferasa (ALT, EC.2.6.1.2) es una enzima cuya estabilidad a corto plazo (hasta siete días) ha sido estudiada por múltiples autores, aunque con resultados discordantes, lo que impide establecer una ecuación de estabilidad consenso [3]. Algunos estudios indican que no hay pérdida significativa de estabilidad en sangre total [6–8] o en suero a 4 °C [9–11], otros indican que incluso hay un aumento de concentración catalítica [12, 13], tanto en sangre total como en suero/plasma. La mayoría, sin embargo, indican un descenso de concentración catalítica a temperatura ambiente (22–25 °C) [9, 14], refrigerado [15] y a −20 °C [9, 14, 16].

Considerando la variabilidad de resultados entre los estudios previos y con el objetivo de integrar variables que pueden influir en la estabilidad y establecer un diseño estandarizado, se decidió realizar un estudio multicéntrico de estabilidad de alanina aminotransferasa (ALT) en ocho hospitales, con cuatro modelos experimentales diferentes y cuatro plataformas analíticas. Como variables con efecto en la estabilidad se incluyeron el tiempo, la temperatura y la concentración catalítica inicial de la enzima.

## Materiales y métodos

Se realizó un estudio multicéntrico en ocho hospitales españoles, para el que se utilizaron muestras de suero de pacientes con concentración catalítica inicial conocida de ALT, conservadas durante siete días a dos temperaturas diferentes (ambiente y refrigerada) y siguiendo cuatro modelos experimentales:

– Modelo 1: se seleccionaron aproximadamente 20 muestras de suero (cinco muestras con ALT>400 U/L (>6.67 μkat/L), cinco muestras con 200 < ALT <400 U/L (3.33 < ALT<6.67 μkat/L), cinco muestras con 50 <ALT <200 U/L (0.83 <ALT <3.33 μkat/L) y cinco muestras con ALT<50 U/L (<0.83 μkat/L). Para cada una de ellas se realizaron diez alícuotas en tubos Eppendorf. La mitad de esas alícuotas (cinco por paciente) se mantuvo a temperatura ambiente, mientras que la otra mitad se refrigeró. Todas ellas se taparon y se protegieron de la luz, y se mantuvieron sin agitación. Una alícuota de cada grupo de cada paciente se congeló a −70 °C en los siguientes tiempos: 0, 1, 2, 3 y 7 días.
  En el octavo día se descongelaron todas las alícuotas y se analizaron en la misma serie analítica. Las muestras con menor concentración catalítica de ALT se analizaron primero para minimizar la contaminación.

– Modelo 2: se seleccionaron aproximadamente 40 muestras de suero: diez muestras con ALT>400 U/L (>6.67 μkat/L), diez muestras con 200 <ALT <400 U/L (3.33 <ALT <6.67 μkat/L), diez muestras con 50 <ALT <200 U/L (0.83 <ALT <3.33 μkat/L) y diez muestras con ALT<50 U/L (<0.83 μkat/L). Inmediatamente después del primer análisis, los tubos primarios se sellaron. La mitad de ellos se refrigeró y la otra mitad se mantuvo a temperatura ambiente, sin luz ni agitación. Se fueron tomando alícuotas de cada tubo para analizar en los tiempos 0 (repetición del valor inicial), 1, 2, 3 y 7 días. En cada caso, el resto de muestra permaneció en el tubo y se tapó inmediatamente.
  En el momento de cada análisis se analizaron primero las muestras de menor concentración catalítica de ALT para minimizar la contaminación.

– Modelo 3: se seleccionaron aproximadamente 200 muestras de suero: 50 muestras con ALT>400 U/L (>6.67 μkat/L), 50 muestras con 200 <ALT <400 U/L (3.33 <ALT <6.67 μkat/L), 50 muestras con 50 <ALT <200 U/L (0.83 <ALT <3.33 μkat/L) y 50 muestras con ALT<50 U/L (<0.83 μkat/L). Inmediatamente tras el primer resultado, se refrigeró la mitad de los tubos primarios mientras que la otra mitad se mantuvo a temperatura ambiente. Todos permanecieron tapados, protegidos de la luz y sin agitación.
  A partir de la fecha de recogida, cada día se seleccionaron 14 tubos de temperatura ambiente y 14 refrigerados y se reanalizaron durante el turno de trabajo habitual del laboratorio.

– Modelo 4: se seleccionaron aproximadamente 20 muestras de suero: cinco muestras con ALT>400 U/L (>6.67 μkat/L), cinco muestras con 200 <ALT <400 U/L (3.33 <ALT <6.67 μkat/L), cinco muestras con 50 <ALT <200 U/L (0.83 <ALT <3.33 μkat/L) y cinco muestras con ALT<50 U/L (<0.83 μkat/L). En las primeras dos horas tras centrifugación se prepararon cuatro *pools* (uno para cada rango de concentración catalítica de ALT), y para cada *pool* se prepararon diez alícuotas en tubos Eppendorf; cinco de las cuales se mantuvieron a temperatura ambiente, y las otras cinco refrigeradas. Todas ellas se taparon, se protegieron de la luz y se mantuvieron sin agitación. Una alícuota de cada grupo se congeló a −70 °C en los siguientes tiempos: 0, 1, 2, 3 y 7 días.
  En el octavo día, todas las alícuotas se descongelaron a la vez y se analizaron por quintuplicado en la misma serie analítica.

Cada modelo se realizó en diferentes laboratorios, utilizando distintas plataformas analíticas (Tabla 1). Se estableció una temperatura controlada de 20–24 °C para incubaciones a temperatura ambiente y 4–8 °C para refrigeración.

**Tabla 1:** Características analíticas de las plataformas analíticas utilizadas en los diferentes estudios experimentales.

Modelo experimental	Plataforma analítica	Metodología (ensayo ALT)	Número de muestras analizadas
1	AU (Beckman Coulter)	NADH con PyP	156
	Architect c16000 (Abbott)	NADH sin PyP	160
	Advia 2400 (Siemens)	NADH sin PyP	160
2	AU (Beckman Coulter)	NADH con PyP	160
	Advia 2400 (Siemens)	NADH sin PyP	96
	Cobas c702 (Roche)	NADH sin PyP	194
3	Advia 2400 (Siemens)	NADH sin PyP	242
4	Cobas c702 (Roche)	NADH sin PyP	80
	Architect c16000 (Abbott)	NADH sin PyP	160

PyP, piridoxal-5'-fosfato; NADH, nicotinamida adenina dinucleótido.

Para evitar la influencia de variables adicionales, las muestras se protegieron de la luz al máximo, se conservaron tapadas o selladas y sin agitación. La calidad de los resultados se aseguró mediante los controles internos habituales para cada laboratorio. Se excluyeron muestras hemolizadas o lipémicas.

### Análisis estadístico

Se utilizó la desviación porcentual (DP) respecto al valor inicial como estadístico básico [(concentración catalítica inicial – concentración catalítica final]/concentración catalítica inicial × 100) y se calcularon las ecuaciones de estabilidad que relacionan la variación de la DP respecto al tiempo de acuerdo con la recomendación de la SEQC^ML^ mediante regresión lineal por el método de mínimos cuadrados [1]. El modelo se forzó a pasar por el origen de coordenadas, ya que a tiempo 0 la pérdida de estabilidad ha de ser cero. Se calculó el intervalo de confianza al 95% para la pendiente. La inspección visual de los resultados sugirió un ajuste lineal de primer grado, más fácil de interpretar, con lo cual los modelos presentados son siempre de la forma:


DP=β×tiempo[días]

(*β* = variación diaria porcentual de la concentración catalítica de ALT).

Para cada ecuación de estabilidad se presenta el nivel de significación del test de contraste (p) y la bondad del ajuste (r de Pearson).

Para evaluar el efecto individual de cada uno de los factores (temperatura, concentración catalítica inicial de ALT, modelo experimental y plataforma analítica) se realizó una regresión lineal múltiple con inclusión de variables predictoras por pasos. Se fijó un error *α* de 0.05 para la inclusión de variables.

En el caso del efecto de la plataforma analítica o la concentración catalítica inicial se efectuó un estudio de ANOVA univariante respecto a la DP con los resultados en el día 7 del estudio. La diferencia individual entre categorías se estudió mediante el test de Diferencias menos significativas DMS

En este estudio se utilizaron muestras de desecho del laboratorio, por lo que no se solicitó consentimiento específico a los pacientes. Todos los datos utilizados fueron previamente anonimizados.

## Resultados

Las características analíticas de cada modelo experimental se presentan en la Tabla 1, junto con el número de muestras analizadas.

De forma global, se observa un claro efecto de la temperatura sobre la estabilidad de la ALT en suero, con una disminución de 4.77%/día a temperatura ambiente y cercana al 0.94%/día en refrigeración. En la Tabla 2 se describen las ecuaciones de estabilidad obtenidas por cada uno de los laboratorios participantes, separando los estudios a temperatura ambiente y refrigerados.

**Tabla 2:** Variación de la concentración catalítica de ALT con el tiempo. Ecuaciones de estabilidad.

Modelo	Plataforma	Refrigerado (n = 704)				Temperatura ambiente (n = 704)			
		p-Value	r	*β*	IC 95% *β*	p-Value	r	*β*	IC 95% *β*
1	Advia	0.001	0.661	−0.902	Entre −1.393 y –0.410	<0.001	0.947	−3.87	Entre −4.504 y –3.248
1	Architect	<0.001	0.500	−1.117	Entre −1.550 y –0.683	<0.001	0.903	−8.742	Entre −9.673 y –7.811
1	AU	<0.001	0.677	0.695	Entre −0.865 y –0.525	0.001	0.679	−3.384	Entre −5.017 y –1.590
2	AU	<0.001	0.752	−1.251	Entre −1.496 y –1.005	<0.001	0.942	−5.199	Entre −5.613 y –4.785
2	Cobas	<0.001	0.870	−1.607	Entre −1.796 y –1.417	<0.001	0.908	−5.824	Entre −6.353 y –5.294
2	Advia	<0.001	0.626	−1.321	Entre −1.783 y –0.858	<0.001	0.829	−5.048	Entre −6.096 y –4.000
3	Advia	<0.001	0.345	−0.605	Entre −0.902 y –0.307	<0.001	0.822	−2.189	Entre −2.463 y –1.915
4	Cobas	0.506	0.107	0.110	Entre −0.222 y 0.442	<0.001	0.939	−2.001	Entre −2.250 y –1.752
4	Architect	0.001	0.720	−1.025	Entre −1.569 y –0.481	<0.001	0.855	−5.609	Entre −7.484 y –3.733
Total		<0.001	0.564	−0.941	Entre −1.051 y –0.831	<0.001	0.832	−4.772	Entre −5.025 y –4.518

p, nivel de significación; r, bondad del ajuste (r de Pearson); *β*, variación diaria porcentual de la concentración catalítica de ALT.

**Figura 1:**
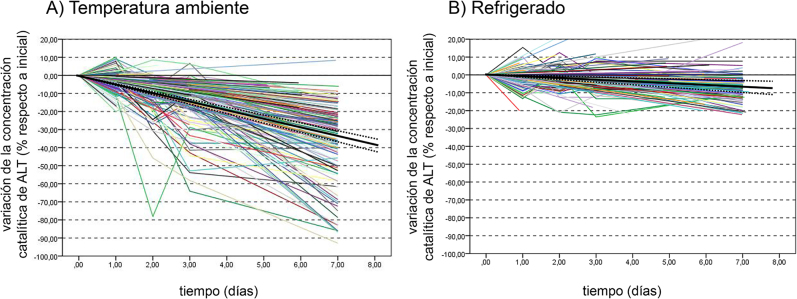
Efecto de la temperatura y el modelo de estudio. Datos individuales de los pacientes, y regresión lineal (línea negra continua) con IC 95% para la pendiente (líneas punteadas). (A) Estudios a temperatura ambiente. (B) Estudios en refrigeración.

La variabilidad interindividual en la pérdida de estabilidad es significativamente superior en aquellas muestras conservadas a temperatura ambiente (Figura 1). La observación de las gráficas sugiere un modelo general de disminución de concentración catalítica lineal con el tiempo en todos los modelos experimentales y plataformas analíticas. La mejora del coeficiente R^2^ con un ajuste cuadrático o cúbico no es significativa ni a temperatura ambiente (0.545 para el ajuste lineal frente a 0.547 para el cuadrático y 0.558 para el cúbico) ni refrigerada (0.165 frente a 0.166 y 0.189).

**Figura 2:**
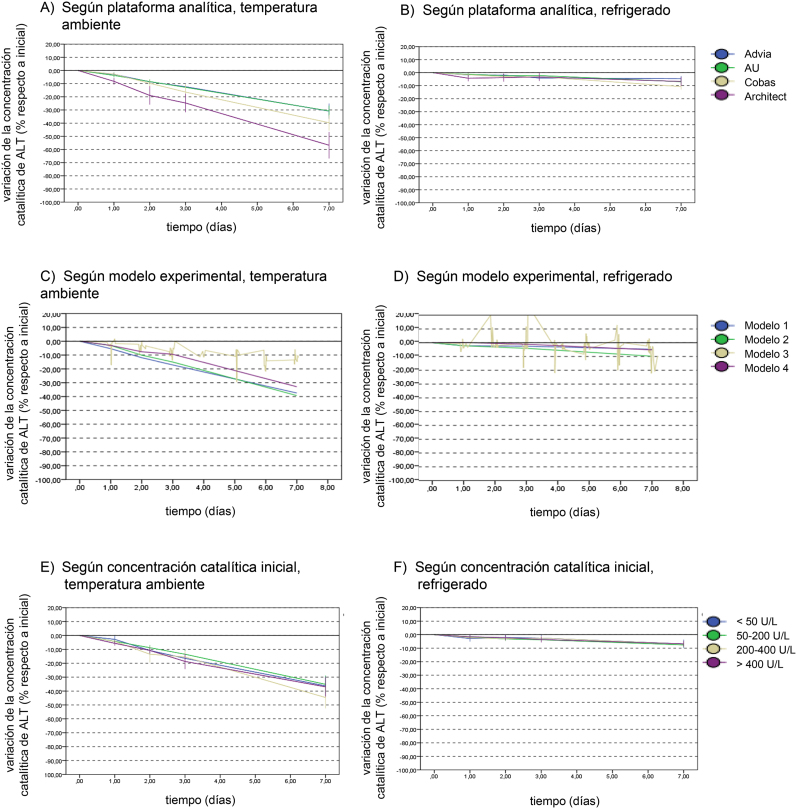
Variación de la concentración catalítica de ALT con el tiempo en función de diferentes variables. (A, B) Plataforma analítica, (C, D) modelo experimental, (E, F) concentración catalítica inicial. Se presenta como gráfico de líneas uniendo las medias para cada tiempo estudiado acompañadas del intervalo de confianza para la media al 95%.

En la Figura 2 se presenta la variación de la concentración catalítica de ALT con el tiempo en función de las variables que se han contemplado en nuestro estudio, como el modelo experimental (2A), la concentración catalítica inicial de ALT (2B) y la plataforma analítica (2C).

En el estudio de regresión lineal múltiple, se introdujeron las cinco variables principales por pasos (por orden: tiempo, temperatura, plataforma analítica, modelo experimental y concentración catalítica inicial), considerando significativo un incremento de la probabilidad de F de 0.05. Se observó que todas las variables tienen un efecto significativo a la hora de determinar la variación de concentración catalítica de ALT, excepto el modelo experimental. La ganancia de explicación del modelo aumenta desde R^2^ = 0.448 con el tiempo como único predictor a R^2^ = 0.483 si se incorpora la temperatura, R^2^ = 0.594 incluyendo el método y R^2^ = 0.602 incluyendo la concentración catalítica inicial, siendo esta la variable con menor peso.

En el caso de la diferencia entre plataformas analíticas observada se realizó un test de ANOVA considerando las medias de las DP a los siete días entre los diferentes equipos, incluyendo todos los resultados (a temperatura ambiente y refrigerado). Se observa una diferencia significativa entre equipos (ANOVA p<0.01) con una mayor pérdida de estabilidad en Architect-Abbott (−31.8%), seguido de Cobas-Roche (−26.3%), AU-Beckman (−18.4%) y Advia-Siemens (−14.5%). El test de (DMS) muestra diferencias significativas entre los equipos Abbott o Cobas frente a los AU o Advia. También se observó diferencia entre los modelos de estudio (ANOVA p<0.01), principalmente entre el modelo 3 (−8.8%) y los demás (−20.7% para el 1, −24.8% para el 2 y −19% para el 4).

En el caso de concentración catalítica inicial, el efecto encontrado en el modelo de regresión múltiple no se observa con los resultados a los 7 días (ANOVA p = 0.430), lo que se corresponde con la escasa diferencia observada en las gráficas.

## Discusión

En este estudio multicéntrico se ha evaluado la influencia de diferentes variables sobre la variación de la concentración catalítica de ALT con el tiempo. En todos los diseños experimentales se ha observado una disminución de la concentración catalítica con el tiempo, que apoya las observaciones descritas en algunas publicaciones [3, 17, 18], aunque contradice a otros autores que no encontraron variaciones [19–21] o incluso que describen aumentos de la concentración catalítica de esta enzima [22].

De acuerdo con nuestros resultados, se observa una influencia muy importante de la temperatura en la estabilidad, siendo ésta cinco veces inferior a temperatura ambiente que en muestras refrigeradas. La mayor pérdida de estabilidad a temperatura ambiente concuerda con lo observado de manera general [22, 23]

Hasta donde sabemos, este es el primer estudio que evalúa la influencia de variables no habituales en la estabilidad de la ALT, como son la concentración catalítica inicial, la plataforma analítica y el modelo experimental [7, 9, 11].

Los estudios previos que han abordado la estabilidad de ALT no valoraron diferentes concentraciones catalíticas, especialmente a niveles altos. El hecho de que muchos estudios solamente incluyeran muestras de individuos con concentraciones catalíticas inferiores o dentro del intervalo de referencia podría suponer una limitación [8, 10, 13, 14, 16], ya que en muchos casos, la propia variabilidad analítica podría enmascarar las variaciones debidas a la pérdida de estabilidad. En nuestro estudio se han incluido grupos de concentración catalítica distribuidos a lo largo del intervalo analítico y con mayor utilidad clínica, desde aquellos inferiores a 50 U/L (0.83 μkat/L) a otros superiores a 400 U/L (3.33 μkat/L). En el estudio multivariante se observa una ligera influencia, con mayor inestabilidad en las muestras con mayor concentración catalítica inicial, aunque este efecto no parece mantenerse a los 7 días, lo que podría sugerir que el efecto se deba realmente a la mayor imprecisión de los métodos de ALT en muestras con baja concentración catalítica.

En el mismo estudio se incluyeron cuatro plataformas analíticas diferentes. Se observó que la pérdida de estabilidad cuando se utilizan los analizadores Architect o Cobas es superior que para los analizadores Advia o AU. La mayor pérdida de estabilidad se obtiene en el analizador Architect. Una justificación a este hallazgo podría ser la diferente composición de los reactivos que utilizan, y que la adición de piridoxal-5’-fosfato (PyP) como coenzima necesario para la reacción mejore la estabilidad del método. Los métodos que no adicionan PyP dependen de la presencia de PyP endógeno en la muestra. Es conocida la degradación de este en relación a la exposición lumínica y la necesidad de refrigerar la muestra para su conservación. La relación entre la disminución del PyP y la concentración catalítica de ALT es una suposición que deberá ser investigada en próximos estudios.

Por su parte, en nuestro estudio planteamos diferentes modelos experimentales para estudiar la estabilidad. Recientemente se han publicado una recomendación de la SEQC^ML^ sobre cómo realizar estudios de este tipo, en los que se enfatiza la importancia de describir y calcular una ecuación o perfil en lugar de definir un único punto o límite de estabilidad [1]. A partir de las ecuaciones de estabilidad, cada laboratorio puede establecer sus propios límites según las especificaciones que se haya fijado.

El modelo experimental a seguir para obtener resultados fiables es de gran relevancia. En nuestro estudio, cada uno de los modelos propuestos tiene ventajas e inconvenientes, tanto a nivel de practicabilidad para realizar el estudio, como a nivel de interpretación de resultados. Por ejemplo, los modelos 1 y 4 se basan en que la congelación de las muestras a −70 °C no implica pérdida de estabilidad. Así, el modelo 2 sería el recomendado si no se pudieran congelar muestras o si ello implicara variación en la concentración catalítica, aunque deberían haberse incluido materiales de control del mismo lote en cada serie para valorar posibles sesgos.

El modelo 1 es el más parecido al que se describe en el reciente protocolo de estabilidad [1], aunque una limitación de los modelos 1, 2 y 3 es haber realizado las determinaciones de forma simple, lo cual impide detectar valores aberrantes. Las repeticiones por quintuplicado del modelo 4 permiten descartar estos valores y disminuyen la imprecisión de los resultados. Sin embargo, la principal limitación de este modelo es el bajo número de muestras que se utilizan (cuatro *pools*). A pesar de las múltiples diferencias entre los cuatro modelos experimentales propuestos, no se han observado diferencias significativas en el modelo de regresión múltiple, aunque sí se observan diferencias a los 7 días. Un posible efecto de confusión es el hecho de que el modelo 3 es el único que no realiza las determinaciones en tiempos fijos y que solo se realizó por uno de los laboratorios debido a su mayor dificultad técnica. El equipo utilizado en este caso (Advia Siemens), fue junto a AU Beckman el que menor pérdida de estabilidad presentó, por lo que podría justificar la diferencia frente a los otros modelos.

Las principales limitaciones de nuestro estudio radican en el reducido número de plataformas analíticas estudiadas y las pocas repeticiones en alguna de ellas. Además, en alguno de los modelos experimentales se incluyó un bajo número de muestras, lo cual dificulta el poder obtener conclusiones robustas. En futuras investigaciones se deberían contemplar variables adicionales que pueden tener influencia en la concentración catalítica de ALT o en la concentración de PyP en la muestra y que están presentes en la práctica rutinaria de los laboratorios clínicos, como el contacto con el aire o la exposición a la luz.

Comparando los resultados individuales de cada paciente agrupados por todas estas variables de influencia, se ha observado una gran variabilidad interindividual en la estabilidad de la ALT. Este fenómeno pone de manifiesto la dificultad y las limitaciones a la hora de establecer un límite de estabilidad único de una determinada magnitud para toda la población bajo unas determinadas condiciones de trabajo. De esta manera, hay individuos con una pérdida más rápida de estabilidad (aquellos con pendientes mayores en la Figura 1), para los cuales se aceptarían algunos resultados como “dentro de estabilidad” cuando realmente su muestra ya no es estable. Lo contrario pasaría con los individuos con concentración catalítica de ALT muy estable en el tiempo, para los cuales se podrían conservar las muestras durante periodos superiores. Este efecto podría en parte deberse a la presencia de isoenzimas termosensibles, como se sugiere por ejemplo para la lactato deshidrogenasa [23].

Se podría hipotetizar que este fenómeno ocurre también en muchas otras magnitudes de interés clínico, y que la simplificación de la estabilidad en un solo valor temporal o una sola ecuación tiene limitaciones importantes, a pesar de ser la mejor herramienta de que disponen los laboratorios clínicos para valorar la estabilidad.

Nuestro estudio pone de manifiesto la necesidad de abordar de forma integral las diferentes variables que pueden afectar a la estabilidad de una magnitud bioquímica, algunas de ellas con poca relevancia en el caso de la ALT (concentración catalítica inicial o modelo experimental), y otras con gran trascendencia (temperatura de conservación o plataforma analítica). La representación gráfica de todas las muestras de individuos incluidos en el estudio permite valorar visualmente la variabilidad interindividual en la pérdida de estabilidad, y la definición de una recta de estabilidad puede ser útil para que cada laboratorio defina sus límites de estabilidad en función de sus propias especificaciones.
